# Development, characterization, and hematopoietic differentiation of Griscelli syndrome type 2 induced pluripotent stem cells

**DOI:** 10.1186/s13287-021-02364-z

**Published:** 2021-05-13

**Authors:** Gülen Güney-Esken, Özgür Doğuş Erol, Burcu Pervin, Gülben Gürhan Sevinç, Tamer Önder, Elif Bilgiç, Petek Korkusuz, Ayşen Günel-Özcan, Duygu Uçkan-Çetinkaya, Fatima Aerts-Kaya

**Affiliations:** 1 Graduate School of Health Sciences, Department of Stem Cell Sciences, Hacettepe University, Sıhhiye, 06100 Ankara, Turkey; 2 Center for Stem Cell Research and Development (PEDI-STEM), Hacettepe University, Sıhhiye, 06100 Ankara, Turkey; 3 School of Medicine, Research Center for Translational Medicine, Koç University, Istanbul, Turkey; 4 Faculty of Medicine, Department of Histology and Embryology, Hacettepe University, Sıhhiye, 06100 Ankara, Turkey; 5 Faculty of Medicine, Department of Pediatrics, Division of Hematology, Hacettepe University, Ankara, Turkey; 6 Laboratory Animals Research and Application Center (HUDHAM), Hacettepe University, Sıhhiye, 06100 Ankara, Turkey

**Keywords:** Griscelli syndrome type 2, Bone marrow, Mesenchymal stromal cells, Hematopoietic stem cells, Induced pluripotent stem cells

## Abstract

**Background:**

Griscelli syndrome type 2 (GS-2) is a rare, autosomal recessive immune deficiency syndrome caused by a mutation in the *RAB27A* gene, which results in the absence of a protein involved in vesicle trafficking and consequent loss of function of in particular cytotoxic T and NK cells. Induced pluripotent stem cells (iPSC) express genes associated with pluripotency, have the capacity for infinite expansion, and can differentiate into cells from all three germ layers. They can be induced using integrative or non-integrative systems for transfer of the Oct4, Sox2, Klf4, and cMyc (OSKM) transcription factors. To better understand the pathophysiology of GS-2 and to test novel treatment options, there is a need for an in vitro model of GS-2.

**Methods:**

Here, we generated iPSCs from 3 different GS-2 patients using lentiviral vectors. The iPSCs were characterized using flow cytometry and RT-PCR and tested for the expression of pluripotency markers. In vivo differentiation to cells from all three germlines was tested using a teratoma assay. In vitro differentiation of GS-2 iPSCs into hematopoietic stem and progenitor cells was done using Op9 feeder layers and specified media.

**Results:**

All GS-2 iPSC clones displayed a normal karyotype (46XX or 46XY) and were shown to express the same *RAB27A* gene mutation that was present in the original somatic donor cells. GS-2 iPSCs expressed SSEA1, SSEA4, TRA-1-60, TRA-1-81, and OCT4 proteins, and *SOX2*, *NANOG*, and *OCT4* expression were confirmed by RT-PCR. Differentiation capacity into cells from all three germ layers was confirmed using the teratoma assay. GS-2 iPSCs showed the capacity to differentiate into cells of the hematopoietic lineage.

**Conclusions:**

Using the lentiviral transfer of OSKM, we were able to generate different iPSC clones from 3 GS-2 patients. These cells can be used in future studies for the development of novel treatment options and to study the pathophysiology of GS-2 disease.

**Supplementary Information:**

The online version contains supplementary material available at 10.1186/s13287-021-02364-z.

## Background

Griscelli syndrome type 2 (GS-2) is a rare, inherited, autosomal recessive disease with a frequency of < 1/1,000,000 in neonates and has been shown to occur more often in Middle Eastern countries, such as Turkey, due to the increased rate of consanguineous marriages [1]. The disease is characterized by partial albinism (silver-colored hair, eyebrows, and eyelashes), hepatosplenomegaly, pancytopenia, immune deficiency, and neurological dysfunction [2]. In GS-2, *RAB27A* mutations cause the malfunction of a small GTPase, which plays an important role in vesicular fusion and cellular trafficking [2]. The RAB27A protein is responsible for the peripheral distribution of melanosomes in melanocytes and exocytosis of cytotoxic granules in the cytosol of cytotoxic T cells (CTL) and natural killer (NK) cells. In addition, RAB27A plays an important role in the binding of cytosolic granules to the cell membrane after T cell receptor signal activation [3–5]. GS-2 patients may also display a decrease in NK cell cytotoxicity resulting in impaired and late hypersensitivity [5]. Although in most cases granulocyte and lymphocyte counts are within normal limits, immunoglobulin levels may be decreased or increased. The only curative treatment for GS-2 is hematopoietic stem cell (HSC) transplantation [1, 6–8]. However, in the absence of human leukocyte antigen (HLA)-compatible donors, no other curative treatment options are available, and therefore, new treatment strategies, such as gene therapy, should be explored.

Via exogenous delivery of the transcription factors Oct4, Sox2, Klf4, and cMyc (OSKM), somatic cells can be reprogrammed into induced pluripotent stem cells (iPSCs) [9], which express typical pluripotency markers, such as SSEA-4, TRA-1-60, TRA-1-81, and alkaline phosphatase, and display three germ layer differentiation potential [9]. Although iPSC lines have been developed from various cell types [10], reprogramming efficiency and differentiation capacity of the iPSCs may vary according to cell source or type [11]. Especially in cases where sufficient cells cannot be obtained due to the rarity of the disorder, iPSC models have shown great potential in providing research material to develop new treatments or diagnostics, but also to study the pathophysiology of the diseases [10, 12]. iPSC lines have been generated for a variety of diseases, including childhood hereditary diseases, and are now deposited in and made available through international iPSC biobanks [13–15]. In addition to disease modeling, iPSCs also enable the development and testing of new treatment modalities, such as gene editing.

Here, we aimed to generate iPSC lines from two different somatic cell types, i.e., mononuclear cells (MNCs) and multipotent mesenchymal stromal/stem cells (MSCs), that were obtained from 3 different GS-2 patients. We intended to generate an in vitro model reminiscent of GS-2 that (1) can be used as a platform to test new treatment strategies, such as gene therapy, and (2) will allow us to obtain a better understanding of the direct effects of the RAB27A mutation on differentiation and function and behavior of hematopoietic cells.

## Materials and methods

### Healthy donor and GS-2 bone marrow samples

Bone marrow (BM) samples of 1–5 mL were aspirated from GS-2 patients (*n*=3) and healthy donors (*n*=7) for diagnostic and/or transplantation purposes, after obtaining written informed consent from each donor. Procedures and sampling were approved by the Hacettepe University Non-Interventional Ethical Committee (GO14/424-19).

### Mesenchymal stromal cell isolation and culture

BM-MNCs were isolated using gradient centrifugation (Biocoll, Biochrom, L6115). MNCs were cultured in DMF10 medium, consisting of a mixture of 60% DMEM-LG (Life Technologies, 31885-049) and 40% MCDB-201 (Sigma-Aldrich, M6770), supplemented with 10% fetal bovine serum (FBS, Life Technologies, 10270-106), 1% l-glutamine (Sigma-Aldrich, G3126), and 1% penicillin-streptomycin (Biochrom, 02A2213). The adherent fraction consisting of mesenchymal stromal cells was expanded at 37°C and 5% CO_2_, with medium replacements every 3–4 days. Confluent cultures were passaged with 0.25% trypsin/1 mM EDTA (Life Technologies, 27250-018).

### Characterization of MSCs

For adipogenic differentiation, MSCs were cultured in adipogenic differentiation medium, consisting of DMEM-LG, 10% FBS, 1 μM dexamethasone (Sigma-Aldrich D2915), 60 μM indometacin (Sigma-Aldrich, 17378), 500 μM 3-isobutyl-1-methylxanthine (Sigma-Aldrich, I5879), and 5 μg/mL insulin. After 3 weeks, cells were stained with 2 mg/mL Oil Red O (ORO, Sigma-Aldrich O0625). ORO dye was extracted from the cells using %2 Igepal (NP40, Sigma-Aldrich, I8896) and measured at 496 nm (Tecan Sunrise microplate reader), as previously described [16]. For osteogenic differentiation, MSCs were cultured in an osteogenic differentiation medium, consisting of DMEM-LG, 10% FBS, 100 nM dexamethasone, 10 mM beta-glycerophosphate (Sigma-Aldrich, G9422), and 0.2 mM l-ascorbic acid (Sigma-Aldrich, A92902). To confirm osteogenic differentiation, cells were stained with Alizarin Red S (ARS). Calcium levels were measured using the Quantichrom Calcium Analysis kit (BioAssay Systems, DICA-500), as previously described [16].

### iPSC generation and expansion

To establish iPSC lines, BM-MNCs or MSCs from three GS-2 patients and one healthy donor were transduced for 2 consecutive days with bicystronic (pSIN4-CMV-K2M, Addgene, 21164; pSIN4-EF1α-O2S, Addgene, 21162) or polycystronic lentiviral vectors (pRRL-PPT-SF-OSKM-IRES-idTomPRE), kindly provided by Prof. Dr. Axel Schambach, Hannover Medical School, Germany [17], carrying the reprogramming factors Oct3/4, Sox2, Klf4, and c-Myc. After transduction, MSCs were cultured in DMF10 and MNCs in StemMACS HSC expansion medium (Miltenyi, 130-100-463) supplemented with StemMACS HSC cytokine cocktail (STF, Stem Cell Factor/Thrombopoietin/Flt3-ligand, Miltenyi, 130-100-843) for 7 days. Transduced cells were plated on Matrigel-coated dishes (Corning, 354277), and the culture medium was replaced by StemMACS iPS-Brew XF culture medium (iPS-Brew, Miltenyi, 130-107-086) with 20 ng/mL basic fibroblast growth factor (bFGF, Immunotools, 11343627). Colonies were picked around day 18 after reprogramming, disrupted by microdissection, and re-plated in iPS-Brew/bFGF supplemented with 10 μM Rock inhibitor Y27632 (Miltenyi, 130-103-922).

### Immunophenotyping of MSCs and iPSCs

MSCs were stained with antibodies against CD29 (BioLegend, 303008), CD44 (BioLegend, 338804), CD73 (BioLegend, 344006), CD90 (BioLegend, 328108), CD105 (eBioscience, 12-1057-41), CD166 (BioLegend, 343904), CD34 (eBioscience, 17-0349-42), CD31 (BD Biosciences, 555445), and HLA-DR (BD Biosciences, 307606) in PBN consisting of phosphate-buffered saline (DPBS 1X, Gibco, 14190144), 0.5% bovine serum albumin (BSA, Sigma, A4503), and 0.05% sodium azide (NaN_3_, Merck, 822335) supplemented with 2% human AB serum. MSCs were assessed with a FACSARIA (Becton Dickinson) or BDAccuri (Becton Dickinson) and analyzed with the FACSDiva V6.12 or BD CSampler analysis software. For iPSC characterization, surface antigens were stained with antibodies against SSEA1 (Miltenyi, 130-104-937), SSEA4 (Miltenyi, 130-098-347), TRA-1-60 (Miltenyi, 130-100-347), and TRA-1-81 (Miltenyi, 130-101-410) in MACS Buffer (PBS, 0.5% BSA and 2 mM EDTA). For intracellular staining, cells were permeabilized with 0.2% Triton (Sigma-Aldrich, X100)/0.2% Tween-20 (Sigma-Aldrich, P1379) and stained with anti-Oct3/4 (Miltenyi, 130-105-555) antibody. iPSCs were washed twice with MACS buffer and measured with a flow cytometer.

### RNA isolation, cDNA synthesis, and quantitative real-time PCR

Total RNA was extracted from iPSCs using Qiazol lysis reagent (Qiagen, 79306) and RNeasy plus Mini Kit (Qiagen, 217004). For cDNA synthesis, 1.3 μg of total RNA was used, and cDNA was synthesized using the Transcriptor High Fidelity cDNA Synthesis kit (Roche, 05081963001). RT-PCR assays were performed with the Lightcycler 480 probes Master kit (Roche, 04 707 494 001) using a LightCycler 480 II (Roche). Expression of *OCT4*, *SOX2*, and *NANOG* pluripotency genes was normalized to the expression of the *B2M* gene, and relative gene expression was calculated using the Livak method [18]. Used primer sequences are shown in Table S1.

### Immunofluorescent labeling of iPSCs

To confirm the expression of pluripotency proteins in selected iPSC clones, clones were fixed with 4% paraformaldehyde and permeabilized with 0.2% Triton X100 + 0.2% Tween-20 in PBS for 20 min, at room temperature. Cells were blocked with a blocking buffer consisting of PBN, 1% AB serum, and 1% mouse serum. iPSCs were stained with the following primary antibodies: rabbit-anti-human OCT3/4 (Molecular Probes, A24867), mouse-anti-human SSEA4 (Molecular Probes, A24866), mouse-anti-human TRA-1-60 (Molecular Probes, A24868), rabbit-anti-OCT4A (Cell Signaling Technology, 2840), rat-anti-SOX2 (Cell Signaling Technology, 3579), rabbit-anti-NANOG (Cell Signaling Technology, 4903), rabbit-anti-c-MYC (Cell Signaling Technology, 5605), and rabbit-anti-LIN28A (Cell Signaling Technology, 3695). Incubations were performed for 2 h at room temperature. Cells were with PBN and then incubated with one of the following secondary antibodies: goat-anti-rabbit IgG-Alexa Fluor 488 (Thermo Fisher Scientific, A11008), goat-anti-mouse IgG-Alexa Fluor 488 (Thermo Fisher Scientific, A11029), anti-rat IgG-Alexa Fluor 488 (Molecular Probes, A24876), anti-mouse IgG-Alexa Fluor 488 (Molecular Probes, A24877), goat-anti-rabbit IgG-Alexa Fluor 568 (Abcam, ab150077), or rat-anti-mouse IgG-FITC (BD Biosciences, 553443). Incubations were performed for 1 h at room temperature, in the dark. Cells were stained with DAPI (300 nM, Sigma-Aldrich, D9542) and analyzed using an Olympus fluorescent microscope.

### Mutation analysis of patient cells before reprogramming

*RAB27A* gene mutations were determined in the material of all three GS-2 patients before and after reprogramming. Selected iPSC clones were expanded and all coding domains, as well as exon-intron intersection domains were assessed by next-generation sequencing with a Miseq device (Illumina) and analyzed using the Miseq Reporter (Illumina) software. Aligned “bam” files were analyzed with the IGV 2.3 (Broad Institute) software.

### Karyotyping of iPSCs

For karyotyping, 3–4 drops of Colcemid were added to the culture and incubated for 3 h. iPSC cultures were trypsinized, and hypotonic salt solution was added to the cell pellets. Cells were prefixed with 5–6 drops of fixative, centrifuged, and resuspended in 5 mL fixative. Cells were washed twice, transferred to slides, and stained with Giemsa. A minimum of 10 metaphases was captured and analyzed.

### In vivo teratoma formation and characterization

1 × 10^6^ iPSCs from different clones derived from both healthy donors and GS-2 patients were resuspended in 100 μL Matrigel and injected directly in the left and right gastrocnemius muscle of Balb/c-Rag2^−/−^ mice (*n*=10), kindly provided by Prof. Dr. Gerard Wagemaker, Erasmus Medical Center Rotterdam, The Netherlands [19], after pretreatment with 25 mg/kg Busulfan (Busilfex, Abdi İbrahim). All animal experiments were approved by the local Hacettepe University animal experiments ethical committee (2014/46-03 and 2015/68-10) and performed at the Hacettepe University Laboratory Animals Research and Application Centre. Teratomas were removed after 6–8 weeks of transplantation for histological analysis and infiltrated with paraffin following immersion fixation in 10% buffered formalin, dehydration in graded series of ethanol, and clearing in xylene. Three to five-micrometer-thick hematoxylin/eosin-stained sections were evaluated under a light microscope (Leica DM6B) with an attached digital camera in order to confirm the presence of three germ layer differentiation using the LASX software.

### In vitro hematopoietic differentiation of iPSCs

1 × 10^6^ healthy donor or GS-2 iPSCs were co-cultured with Op9 stromal cells (kindly provided by Prof. Dr. Juan Carlos Zúñiga-Pflücker, Sunnybrook Research Institute, Toronto, Canada [20]) in different HSC differentiation media for 8 days. A total of 7 clones (2 from healthy donor iPSCs, 5 derived from 3 different GS-2 patients) were tested for HSC differentiation potential. HSC differentiation media consisted of serum-free StemMACS HSC expansion medium with 1× hematopoietic expansion cocktail (STF: SCF, TPO, Flt3-ligand), 50 ng/mL BMP4 (Bone Morphogenic Protein 4 (R&D Systems, 314-BP-050), 5 μM ATRA (all-trans retinoic acid, Sigma-Aldrich, R2625) and 1 μM dexamethasone. Differentiated CD34+ HSC were further expanded with StemMACS HSC expansion medium with 1× STF for 1–2 weeks. Immunophenotype of the differentiated cells was measured using anti-CD43 (BioLegend, 343206), anti-CD45 (BD Biosciences, 560976), anti-CD34 (eBioscience, 17-0349-42), and anti-CD38 (BD Biosciences, 555459) antibodies. To assess the colony-forming capacity of HSCs differentiated from iPSC, the cells were cultured in Methocult H4434 classic (Stem Cell Technologies, 04444) for 10–14 days. After counting, colonies were picked and stained with anti-CD45, anti-CD16 (BioLegend, 302012), anti-CD14 (BD Biosciences, 555399), and anti-CD33 (BioLegend, 366608) to confirm myeloid differentiation.

### Transplantation and engraftment of iPSC-derived HSCs

To test the quality of HSCs derived from a healthy donor and GS-2 iPSCs, a total of 15 mice were transplanted with 5 × 10^5^–1 × 10^6^ HSCs derived from healthy donor iPSCs (*n*=3, control group) or GS-2 iPSCs (*n*=12). The HSCs were cultured expanded as explained above, resuspended in 100 μL PBS and transplanted i.v. into immune-deficient Balb/c Rag2^−/−^ mice after pre-treatment with 25 mg/mL busulfan (BU). Mice were assessed for human engraftment by measuring the levels of anti-human CD45, CD10 (BD Biosciences, 347503), CD19 (BD Biosciences, 555413), CD3 (BD Biosciences, 555333), CD4 (BD Biosciences, 555346), and CD8 (BD Biosciences, 555369) in peripheral blood samples at months 1, 2, 3, and 6 after transplantation. Bone marrow and spleen cellularity and human engraftment were assessed following sacrifice of mice from each group at 3 or 6 months after transplantation.

### Statistical analysis

Differences in gene expression levels between somatic cells before reprogramming and generated iPSC clones were determined after normalizing genes of interest with the reference gene. The standard deviation of the mean was calculated, and *T* tests were performed using the Excel software program. *P* values <0.05 were considered significant.

## Results

### Characteristics of GS-2 and healthy donor BM-MSCs

**Fig. 1:**
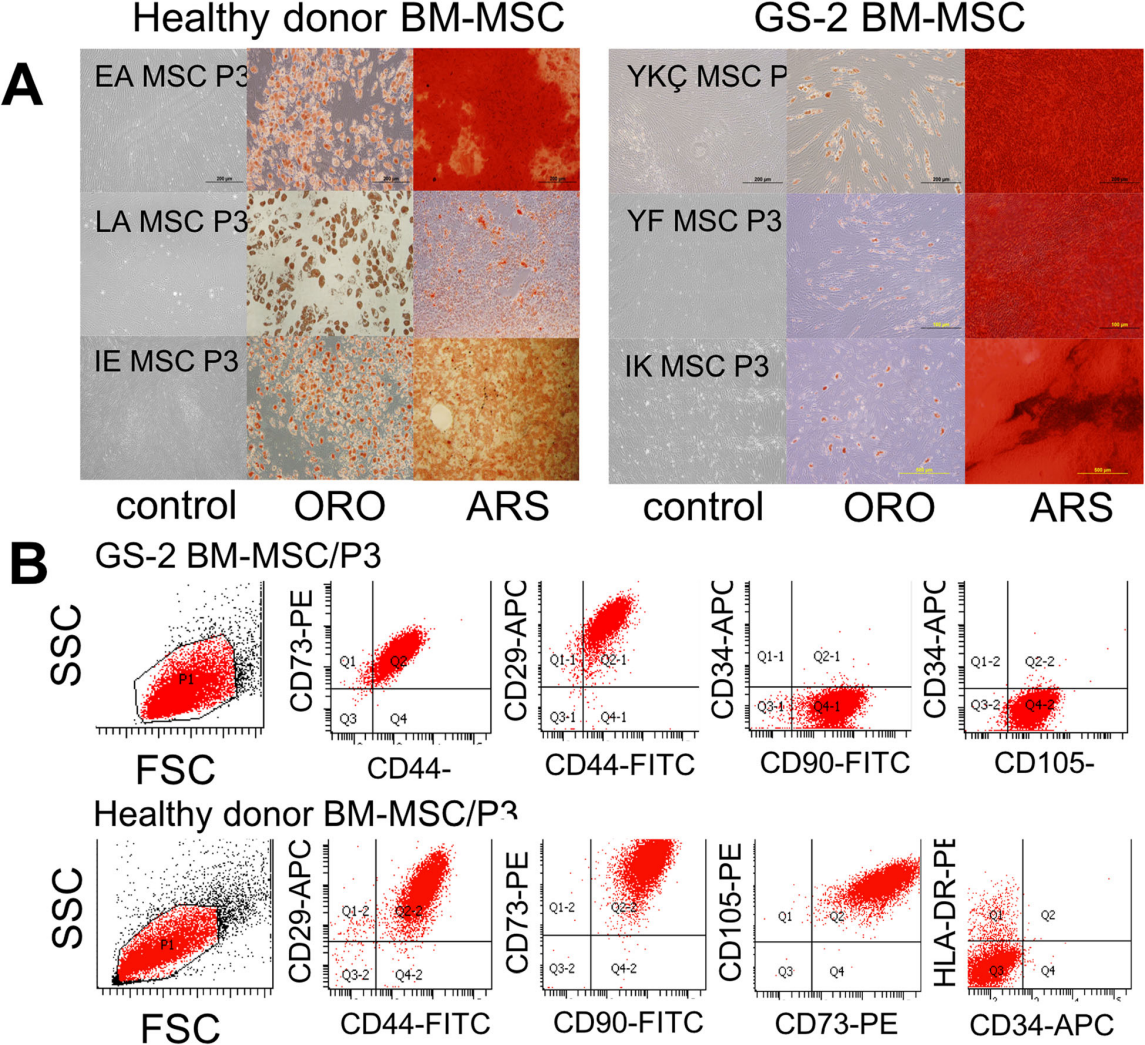
Characterization of a healthy donor and GS-2 BM-MSCs. **a** Morphology before and after differentiation of healthy donor (left, *n*=3) and GS-2 (right, *n*=3) BM-MSCs. MSCs were cultured in control medium (left lines) or adipogenic and osteogenic differentiation media for 21 days and were stained with Oil Red O (midline, ORO) for adipogenic differentiation and Alizarin Red S (right lines, ARS) for osteogenic differentiation. **b** Immunophenotype of a healthy donor and GS-2 BM-MSCs. Dot plots from representative BM-MSC samples of passage 3 GS-2 (upper lane) and healthy control (lower lane) BM-MSCs

Microscopically, MSCs from healthy and GS-2 samples were similar and showed a spindle-shaped morphology (Fig. 1a). No significant differences were found in terms of differentiation capacity (Fig. 1a, Table S2). Both healthy and GS-2 MSCs were highly positive for specific MSC markers CD29, CD44, CD73, CD90, CD105, CD166, and HLA-DR and negative for the endothelial and hematopoietic markers CD31 and CD34 (Fig. 1b, Table S3).

### Lentiviral reprogramming of healthy and GS-2 MNCs and MSCs

**Fig. 2:**
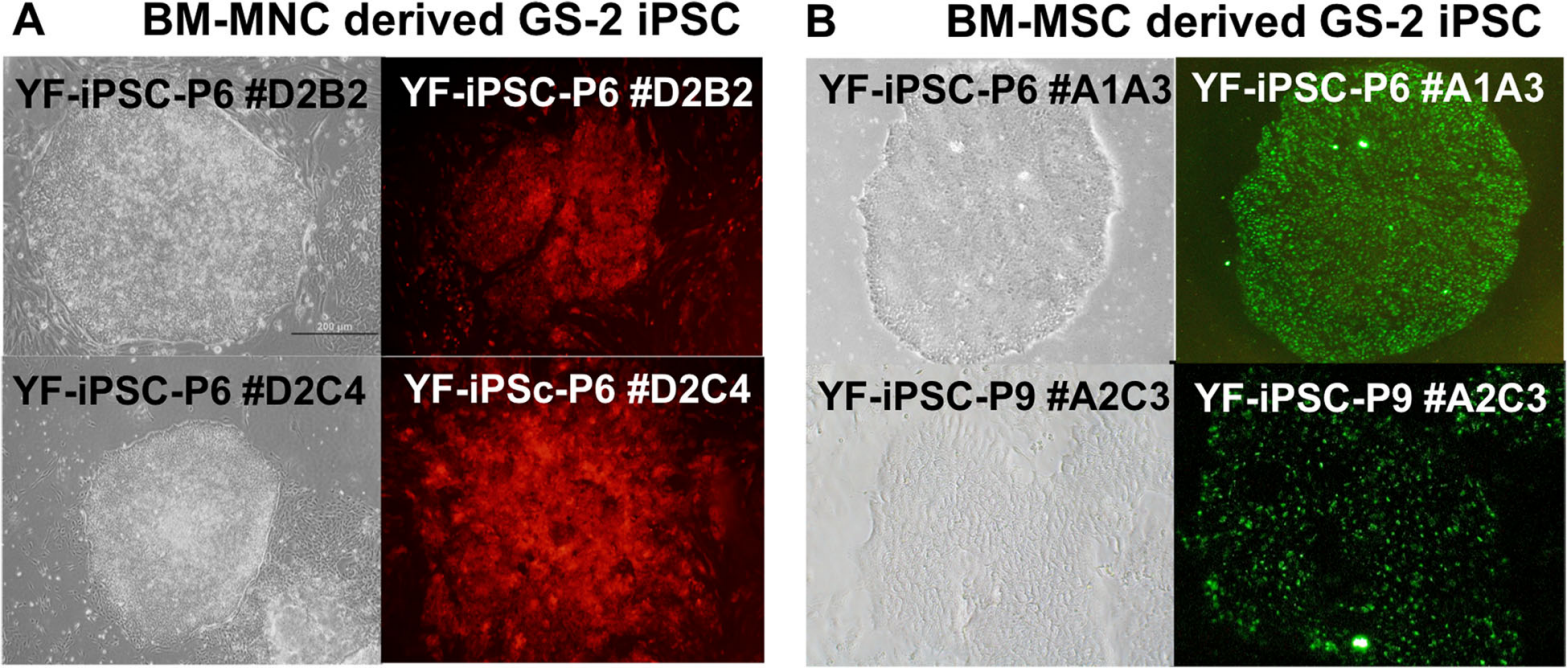
Morphology of BM-MNC- and BM-MSC-derived iPSC clones. **a** Morphology and idTOM (red) expression of BM-MNC derived GS-2 iPSC clones YF #D2B2 and #D2C4. **b** Morphology and TRA-1-60 (green) expression of BM-MSC derived GS-2 iPS cell clones YF #A1A3 and #A2C3. Olympus inverted fluorescent microscope images, ×10 magnification

Eighteen days after reprogramming of healthy donor and GS-2 MNCs, using LV-SF-OSKM, formation of idTOM-positive iPSC-like colonies was observed (Fig. 2a). Passage 3 BM-MSCs from healthy (*n*=1, GP) and GS-2 (*n*=3; YF, IK, YKÇ) donors were reprogrammed using the bicistronic vector for YF and IK and the polycistronic vector for YKÇ, and colonies were collected between 18 and 21 days. At least 5 iPSC clones were generated for each sample, and the best clones were chosen for further use and characterization (Fig. 2b). iPSC clones derived from healthy and GS-2 samples maintained consistent morphology and growth rates for up to 12 passages.

### Characterization of healthy donor and GS-2 iPSC clones

**Fig. 3:**
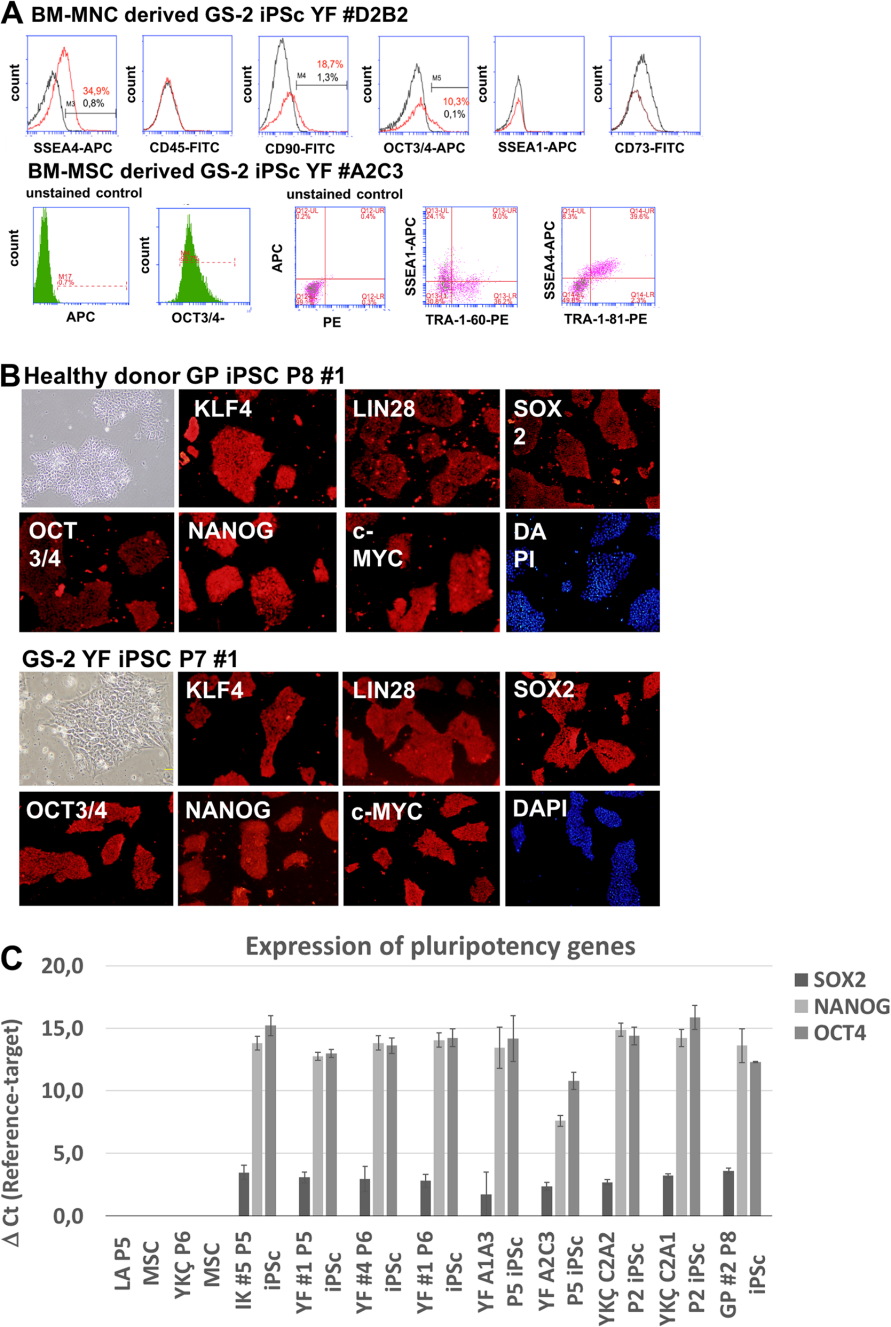
Immunophenotype of BM-MNC- and BM-MSC-derived iPSC clones. **a** Top lane: representative histograms of a GS-2 iPSC clone (YF #D2B2) derived from BM-MNCs. Unstained controls are given as black lines and stained iPSC as red lines. Lower lane: representative histograms and dot plots from a BM-MSC-derived GS-2 iPSC clone (YF #A2C3). **b** Healthy donor iPSc (top) and GS-2 iPSC (bottom) immunofluorescence images, representative samples. Both healthy donor and GS-2 iPSCs show bright expression of pluripotency markers. The nuclei were counterstained with DAPI. **c** Relative gene expression levels of *SOX2*, *NANOG*, and *OCT4* normalized to *B2M* expression. Healthy (GP) and GS-2 (IK, YF, YKÇ) iPSC clones showed increased levels of *SOX2*, *NANOG*, *OCT4* gene expression in comparison with healthy donor (LA) and GS-2 (YKÇ) BM-MSCs (negative controls)

iPSC clones generated from BM-MNCs expressed the pluripotency markers SSEA-4 and OCT3/4, but not the differentiation-related marker SSEA-1. MNC-derived GS-2 iPSC cells did not express MSC or hematopoietic surface markers (CD73, CD45), and CD90 expression was found to be low (Fig. 3a, top lane). BM-MSC-derived healthy donor and GS-2 iPSC clones were highly positive for OCT3/4 and showed positivity for SSEA-1, SSEA-4, TRA-1-60, and TRA-1-81 (Fig. 3a, lower lane). Expression of LIN28, KLF4, c-MYC, OCT3/4, SOX2, and NANOG in healthy donor and GS-2 iPSC clones was further confirmed using immunofluorescent staining (Fig. 3b). All iPSC clones were analyzed for *SOX2*, *NANOG*, and *OCT4* gene expression by RT-PCR. Healthy and GS-2 BM-MSCs were used as negative controls. Healthy donor (LA) and GS-2 (YKÇ) BM-MSC *SOX2*, *NANOG*, and *OCT4* gene expression was low in comparison with the iPSC clones. Increased gene expression of *NANOG*, *OCT4*, and *SOX2* was detected in all iPSC clones tested (Fig. 3c), although not all clones showed a similar increase in gene expression.

**Fig. 4:**
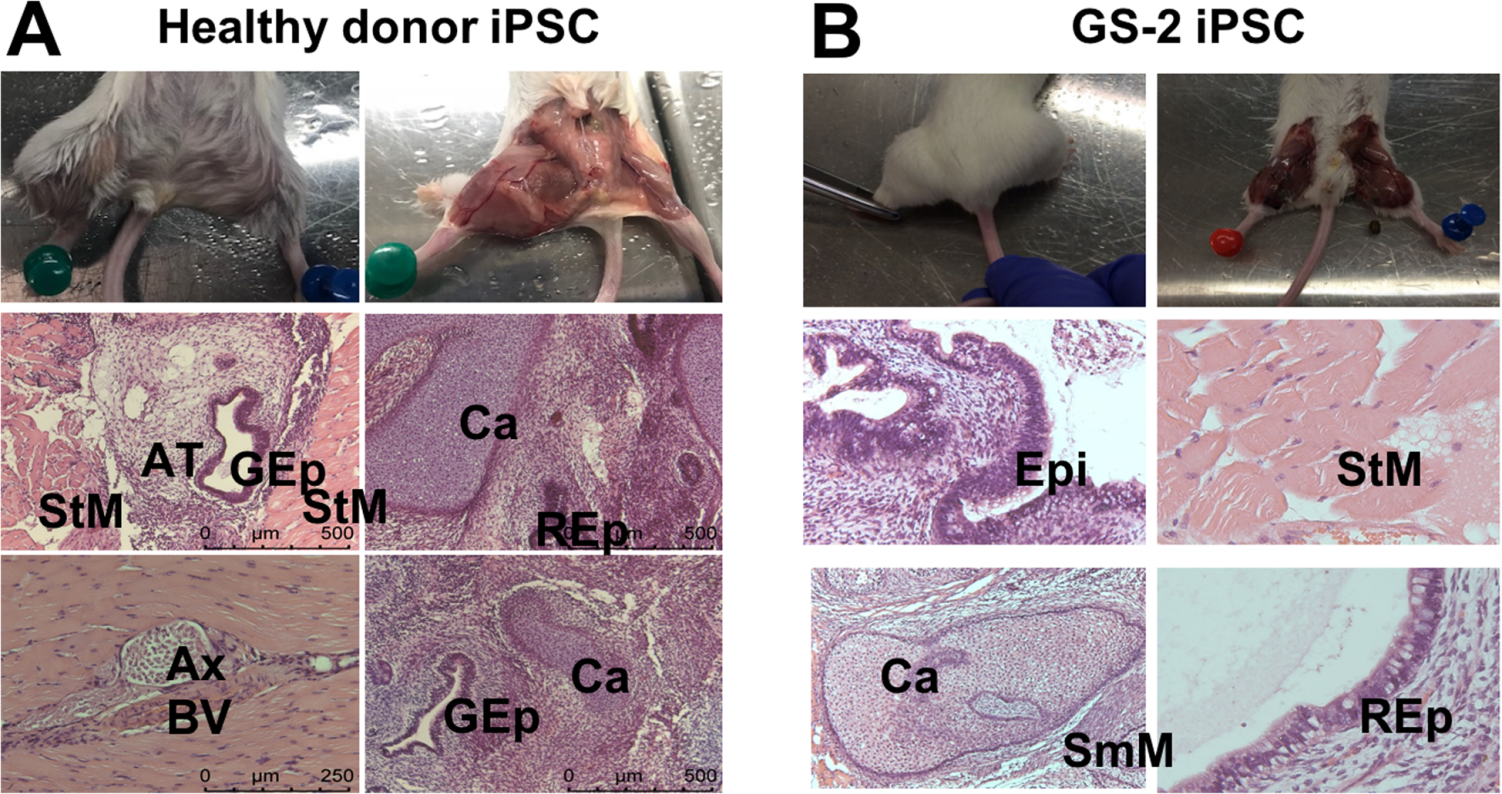
Teratoma capacity of a healthy donor and GS-2 iPSCs. **a** 1 × 10^6^ healthy donor iPSCs were injected intramuscularly in Rag2^−/−^ mice and followed for 6–8 weeks. Mice were sacrificed when palpable tumors appeared. Teratomas were fixed and stained with hematoxylin/eosin. Teratomas reveal the presence of mesodermal striated muscle (SM), adipose tissue (AT), cartilage (Ca), blood vessel (BV), endodermal respiratory (REp) and gastrointestinal (GEp) epithelium, and ectodermal axons (Ax). **b** 1 × 10^6^ GS-2 iPSCs were injected intramuscularly in Rag2^−/−^ mice. Teratomas reveal the presence of mesodermal striated muscle (StM) and smooth muscle (SmM), blood vessel (BV), cartilage (Ca), endodermal respiratory endothelium (RE), and ectodermal epidermis (Epi). Pictures from representative samples

Karyotyping of healthy and GS-2 iPSC clones showed normal karyotypes consisting of 46XX or 46XY chromosomes (Figure S1). Teratoma slides revealed the presence of mesodermal striated and smooth muscle, adipose tissue, cartilage, blood vessel, endodermal respiratory and gastrointestinal epithelia, and ectodermal epidermis and axons histologically (Fig. 4), confirming the potential for differentiation into three germ layers of these iPSC clones.

Mutation analysis of healthy and GS-2 iPSC clones confirmed the absence of *RAB27A* mutations in healthy donor iPSCs and the presence of specific *RAB27A* gene mutations at the same locations in the GS-2 iPSC clones and the original GS-2 samples (Table 1).

**Table 1:** RAB27A gene mutations present in healthy donor and GS-2 iPSC clones

iPSC clones	*RAB27A* (NM_004580)
Healthy donors	
GP/P8/clone #1	Ok
GP/P8/clone #2	Ok
GS-2 patients IPSCs	
IK/P8/clone #5	c.514_518delCAAGC %100
IK/P6/clone #2	c.514_518delCAAGC %100
YF/P6/clone #1	c.148-149delAGinsC %100
YF/P6/clone #4	c.148-149delAGinsC %100
YF/P8/clone #A1A3	c.148-149delAGinsC %100
YF/P8/clone #A2C3	c.148-149delAGinsC %100
YKÇ/P6/clone #C2A1	c.148-149delAGinsC %100
YKÇ/P6/clone #C2A2	c.148-149delAGinsC %100

### In vitro hematopoietic differentiation of healthy and GS-2 iPSCs

**Fig. 5:**
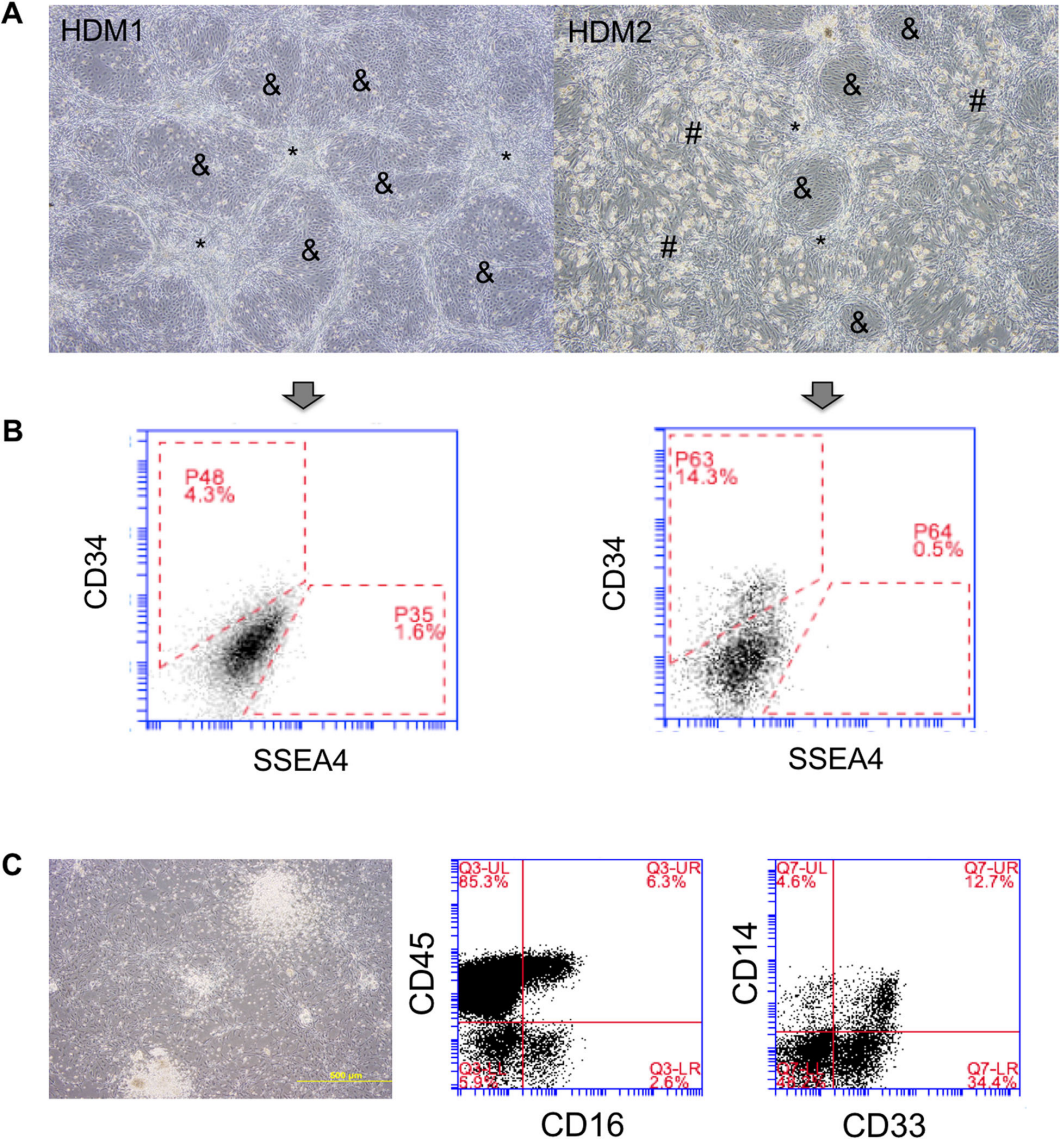
In vitro hematopoietic differentiation of a healthy donor and GS-2 iPSCs. Healthy donor and GS-2 iPSCs were co-cultured with hematopoietic differentiation medium (HDM) 1 (left) and 2 (right) for 9 days on the confluent feeder layers of Op9 cells. For specifics of the media, see the main text. **a** Within 4–5 days, hematopoietic island appeared in separate areas surrounded by stromal Op9 cells. *Unchanged Op9 cells; ^#^differentiated Op9 cells; ^&^islands of differentiating iPSC/HSCs. **b** FACS dot plots of the above co-cultures. **c** Microscopic photograph of colony assays (left) showing prominent white colonies that were confirmed to be CFU-GM by FACS analysis (right)

**Fig. 6.**
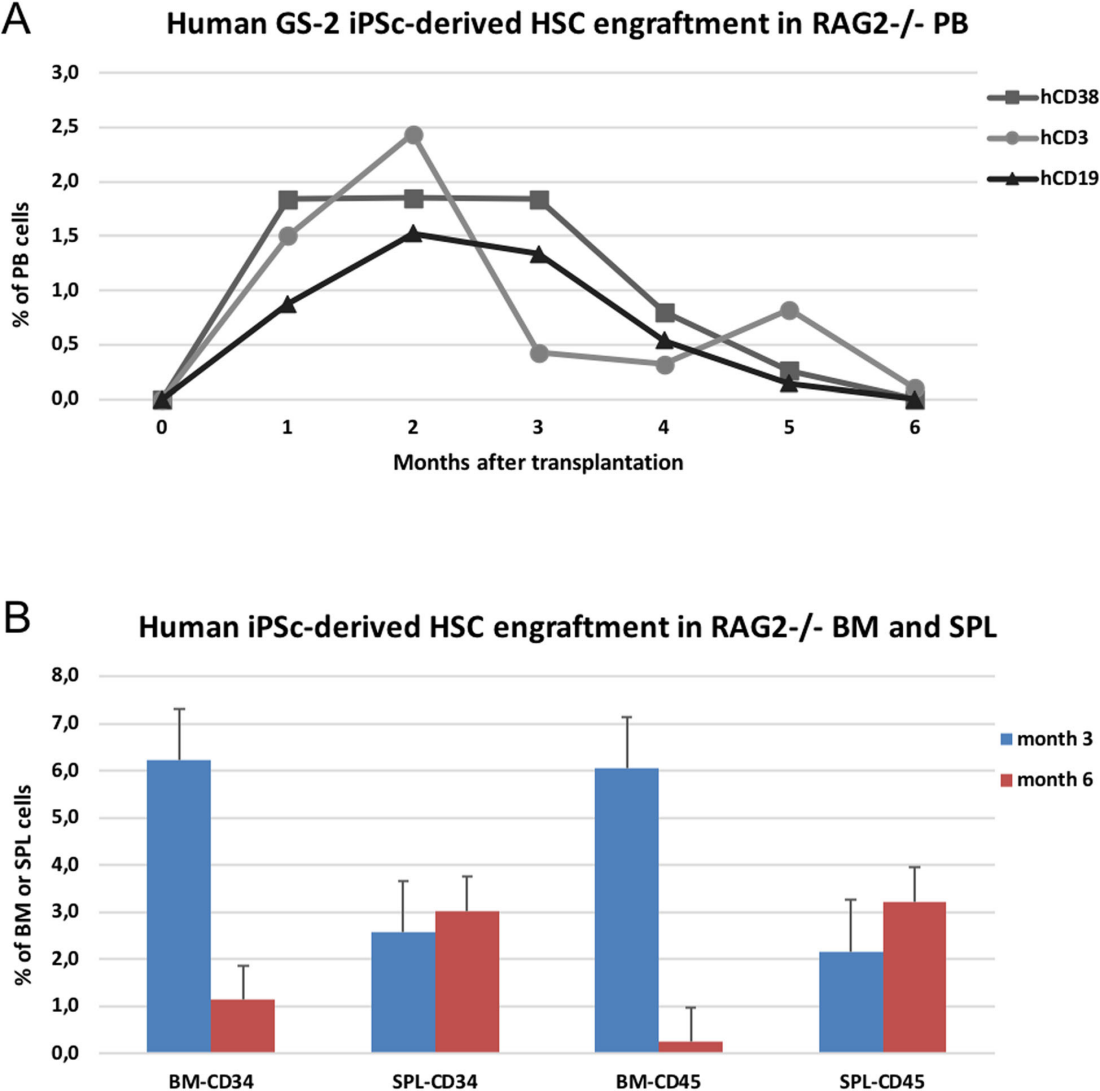
Engraftment of human iPSC-derived HSCs in immune-deficient mice. Immune deficient Rag2^−/−^ mice were pre-treated with busulfan and transplanted with 5 × 10^5^–10^6^ cells obtained from human iPSC-derived HSC cultures per mouse (*n*=15). **a** Peripheral blood samples were collected at 1–6 months after transplant and assessed for the presence of human hematopoietic cells. **b** Groups of mice were sacrificed at month 3 and 6 to assess the presence of human hematopoietic cells in the spleen (SPL) and bone marrow (BM) tissue

iPSCs were co-cultured on Op9 cells with hematopoietic differentiation medium 1 (HDM1: αMEM, 10% FBS-HI, 1% penicillin/ streptomycin, 1% l-glutamine, 5 mM β-mercaptoethanol, 4 mg/mL l-ascorbic acid, 10 μg/mL BMP-4) or hematopoietic differentiation medium 2 (HDM2: StemMACS HSC expansion medium, 1X STF, 10 μg/mL BMP-4). Within 4–5 days after co-culture, islands of hematopoiesis appeared in the cultures (Fig. 5a), and after 9 days of co-culture, cells were assessed for the presence of CD34, CD38, CD45, and/or CD43, a pan-hematopoietic marker, that has been shown to define the earliest hematopoietic progenitors in embryonic stem cell (ESC) cultures [21]. The results showed the presence of up to 14.3% CD34+ in serum-free HDM2 medium, despite considerable adipogenic differentiation of the stromal feeder layer Op9 cells. In contrast, significantly lower levels of CD34+ cells (4.3%) were detected in co-cultures maintained in HDM1 where, interestingly, the morphology of the Op9 cells remained unchanged (Fig. 5b). Not all clones were tested for hematopoietic differentiation potential, but from the 7 clones (2 healthy donor iPSC clones, 5 GS-2 iPSC clones, derived from MNCs and MSCs) tested, with the exception of one GS-2 clone (YF #A2C3), all clones showed expression of CD34+ after 9 days of co-culture with Op9 cells. This particular clone also showed much lower gene expression of *NANOG* and *OCT4* (Fig. 3c) and was most likely only partially reprogrammed. Colony assays performed with iPSC-derived HSCs showed a preference for white colonies, which were confirmed to be mixed CFU-GM colonies expressing CD45 (91.6–92.3%), CD33 (47.1–95.0%), CD16 (9.0–9.1%), and CD14 (12.4–17.5%) markers (Fig. 5c). Optimization of the culture conditions by addition of ATRA and dexamethasone, in combination with further expansion of the iPSC-derived HSCs for an additional 21 days in presence of HSC expansion medium supplemented with 1X STF resulted in the expansion of considerable numbers of CD34+ cells, ranging from 40 to 80% CD34 after 14–21 days of expansion culture (Figure S2). When these cells were transplanted into Rag2^−/−^ mice at a dose of 5 × 10^5^–10^6^ cells/mouse, engraftment was observed at 1 month after transplantation with the appearance of human cells in peripheral blood (PB) samples of mice (Fig. 6a). Maximal engraftment was observed at 2 months after transplantation, after which levels of human cells in murine PB steadily decreased. In addition to the presence of human leukocytes in PB, the presence of CD34+ and CD45+ human hematopoietic cells was also detected in BM and spleen at 3 and 6 months after transplantation (Fig. 6b).

## Discussion

GS-2 syndrome is a rare autosomal recessive immunodeficiency that can only be treated with HSC transplantation. The rarity of the disease makes it difficult to obtain sufficient cell numbers for research and to allow the development of new treatment strategies. The two GS-2 mouse models, i.e., the C3H/HeSn-Rab27a^Ash^/J or “Ashen mouse” [22] and the C57BL/6J-Rab27a^Ash^/J (Rab27a^−/−^) mouse model created by backcrossing into C57BL/6J mice [23], both display an immune deficiency and HLH phenotype similar to GS-2 syndrome in humans. Nonetheless, an in vitro human GS-2 model is needed to develop and test new treatment strategies. Therefore, we aimed to generate several iPSC lines from GS-2 patients with different RAB27A mutations. Here, we show that iPSC clones derived from GS-2 MNCs and MSCs fulfill all the requirements of pluripotent stem cells, including the expression of pluripotency genes and differentiation into cells from all three germ cell layers.

In addition to the detailed characterization of GS-2 iPSCs, we also provide a detailed characterization of GS-2 MSCs and compared the latter with healthy donor MSCs. In terms of “morphology” and immunophenotype, we found no differences between GS-2 and healthy donor MSCs. In addition, GS-2 MSC proliferation and differentiation capacity were similar to healthy donor MSCs. Thus, it appears that the RAB27A mutation does not negatively affect the overall proliferation or differentiation capacity of MSCs.

iPSC clones were successfully derived from both GS-2 MNCs and MSCs using lentiviral vectors, and no differences were observed in terms of morphology, immunophenotype, or expression of pluripotency genes in iPSCs derived from these two somatic cell sources. However, we did notice that using the same concentration of OSKM virus, MNCs showed a tendency for rapid reprogramming, whereas MSCs appeared to be more resistant to reprogramming, requiring a higher MOI. These data are in line with a study showing that cell type may affect reprogramming efficiency [24]. Similar to our results, Wang and colleagues showed that reprogramming of MNCs appeared to be more effective than reprogramming of MSCs [25].

Using different protocols to induce hematopoietic differentiation of the GS-2 iPSC clones, we found that MNC-derived iPSCs more rapidly differentiated towards HSCs, giving rise to up to 17% CD34+ in co-cultures with Op9 cells. In contrast, HSC differentiation from iPSCs derived from GS-2 MSCs proved much more difficult, resulting in much lower percentages of CD34+ cells (ranging from 2 to 4%). These data may indicate that the inherent, epigenetic memory of the iPSCs may influence both the efficacy of reprogramming, but also the differentiation capacity of these cells, at least in vitro. Accordingly, it has been shown previously that iPSCs may preserve their epigenetic memory and that tissue or somatic cell type-specific DNA methylation patterns may remain despite reprogramming, causing a relative preference of these cells to re-differentiate towards their cell type of origin rather than transdifferentiation into another lineage [26, 27].

Choi et al. co-cultured iPSCs with Op9 cells and found similar levels of CD34+ cells [28]. However, these levels leave room for improvement, and many groups are currently working on protocols to increase the efficacy and efficiency of HSC differentiation. For example, ATRA has been shown to increase hematopoietic differentiation potential [29]. In addition, certain molecules, such as WNT pathway inhibitors (CHIR99021) [30, 31], BMP4, and bFGF, have been reported to increase hematopoietic differentiation efficiency [32]. Using different combinations of these factors, we managed to increase the levels of CD34+ cells in vitro. Further culture modifications, including the use of gelatin-coated surfaces, different densities, and/or irradiation of Op9 cells, may further improve hematopoietic differentiation of iPSCs. Alternatively, Cerdan and colleagues performed HSC differentiation using embryonic body formation directly from ESCs and were able to obtain 90% CD45+ cells [33]. iPSC-derived HSCs were transplanted in immune-deficient Rag2^−/−^ mice, and engraftment was followed for up to 6 months. Despite high levels of CD34+ cells obtained in in vitro cultures, we found that human chimerism after the initial engraftment rapidly decreased after 2 months of transplantation. This lack of long-term engraftment may have been caused by many factors, including prolonged in vitro expansion of the CD34+ cells, which may have depleted long-term repopulating cells. Furthermore, the cells were not further sorted or selected for CD34 positivity, and infused cell populations may not have been sufficient to sustain engraftment. Also, in contrast to the Rag2^−/−^/IL2R_γc_^−/−^ mice, the Rag2^−/−^ mice that we used here are not the preferred humanized mouse model due to their remaining NK cell function. In addition, the low-dose preparatory regime used (25 mg/kg busulfan) may not have been sufficient to open up the bone marrow space and suppress remaining immunity. Therefore, engraftment might have been better or could have lasted longer in other humanized models, such as the NOD/SCID-IL2Rγ^−/−^ (NSG) mice and NSG mice transgenic for human hematopoietic growth factors [34]. Lastly, iPSC-derived CD34+ cells may show defects in homing and/or cannot be sustained/maintained in the bone marrow due to the absence of certain surface antigens, including adhesion molecules. It is furthermore conceivable that iPSC-derived CD34+ cells may not have sufficiently long telomeres to support long-term hematopoiesis. Since we cannot rule out that the observed short-term engraftment was caused by the animal model, rather than by the transplanted iPSC-derived HSCs, all these factors should be further investigated. Nevertheless, the data are promising, and using modifications of the current protocols, both in vitro and in vivo, we believe that transplantation and long-term engraftment of iPSC-derived HSCs will be feasible.

## Conclusions

Here, we show that the iPSC clones derived from GS-2 patients fulfill all requirements of true pluripotent stem cells, including the expression of pluripotency genes and the capacity for differentiation into cells of all three germlines. In addition, the ability of GS-2 iPSCs to differentiate in vitro into HSCs and allow, at least short-term, in vivo engraftment underlines the potential of these cells for the development of future treatment modalities. With further optimization of the protocols, these cells could serve as a model to investigate the effects of the RAB27A mutation on HSC differentiation and HSC potential and its role in the impairment of cytotoxic T cells and NK cells. Furthermore, this GS-2 iPSC model can be used to develop new lentiviral or CRISPR/Cas9-based gene therapy strategies for the treatment of Griscelli syndrome type 2.

## Supplementary Information

**Additional file 1: Figure S1.** Karyotype analysis of healthy donor and GS-2 iPSC clones. For karyotyping, iPSC cultures were trypsinized and treated with a hypotonic salt solution. 10 metaphases were captured and analysed. Shown here are the karyotypes of a healthy donor (left, GP) and the 3 GS-2 patient-derived iPSCs (IK, YF, YKÇ). **Figure S2.** Upper panel: iPSCs were co-cultured with Op9 cells in presence of HDM2 medium (StemMACS HSC expansion medium, 1X STF, 10 μg/mL rhBMP-4), resulting in low level expression of CD34. Lower panel: further expansion of iPSC-derived HSCs in StemMACS HSC expansion medium, 1X STF for 21 days resulted in expansion of CD43+, CD34+, CD45+ HSCs. **Table S1.** Primer sequences used for RT-PCR. **Table S2.** Differentiation capacity of healthy donor and GS-2 BM-MSCs. For adipogenic differentiation, MSCs were cultured in DMEM-LG, supplemented with 10% FBS, 1 μM dexamethasone, 60 μM indometacin, 500 μM 3-isobutyl-1-methylxanthine and 5 μg/mL insulin. After 3 weeks, cells were stained with 2 mg/mL Oil Red O (ORO, Sigma-Aldrich O0625). ORO dye was extracted from the cells using %2 Igepal and measured at 496 nm on a microplate reader. For osteogenic differentiation, MSCs were cultured in DMEM-LG, 10% FBS, 100 nM dexamethasone, 10 mM beta-glycerophosphate and 0,2 mM L-ascorbic acid. Calcium levels were measured using the Quantichrom Calcium Analysis kit (BioAssay Systems, DICA-500). **Table S3.** Immunophenotype of healthy donor and GS-2 BM-MSCs. MSCs from healthy donors and GS-2 patients were generally positive for MSC-specific surface antigens (CD29, CD44, CD73, CD90, CD105 and CD166) and negative for hematopoietic and endothelial cell markers. No significant differences were found in expression of cell surface antigens by MSCs between the healthy donors and GS-2 patients.

## Data Availability

Data are made available through supplementary materials. Data not available through the supplementary materials are available from the corresponding author on reasonable request.

## Abbreviations

GS-2: Griscelli syndrome type 2
iPSCs: Induced pluripotent stem cells
OSKM: Oct4, Sox2, Klf4, and cMyc
CTL: Cytotoxic T cells
NK cells: Natural killer cells
HSCs: Hematopoietic stem cells
HLA: Human leukocyte antigens
MNCs: Mononuclear cells
MSCs: Multipotent mesenchymal stromal/stem cells
BM: Bone marrow
FBS: Fetal bovine serum
EDTA: Ethylenediaminetetraacetic acid
ORO: Oil Red O
ARS: Alizarin Red S
bFGF: Basic fibroblast growth factor
DPBS: Dulbecco’s phosphate-buffered saline
BSA: Bovine serum albumin
DAPI: Diamidino-phenylindole
STF: SCF, TPO, Flt3-ligand
BMP4: Bone morphogenic protein 4
ATRA: All-trans retinoic acid
