# Supplementary material for: Development, characterization, and hematopoietic differentiation of Griscelli syndrome type 2 induced pluripotent stem cells
Source: Stem Cell Res Ther. 2021 May 13;12:287. doi: 10.1186/s13287-021-02364-z (PMC8117610; doi:10.1186/s13287-021-02364-z)
Supplement: Supplementary file 1 — Additional file 1: Figure S1. Karyotype analysis of healthy donor and GS-2 iPSC clones. For karyotyping, iPSC cultures were trypsinized and treated with a hypotonic salt solution. 10 metaphases were captured and analysed. Shown here are the karyotypes of a healthy donor (left, GP) and the 3 GS-2 patient-derived iPSCs (IK, YF, YKÇ). Figure S2. Upper panel: iPSCs were co-cultured with Op9 cells in presence of HDM2 medium (StemMACS HSC expansion medium, 1X STF, 10 μg/mL rhBMP-4), resulting in low level expression of CD34. Lower panel: further expansion of iPSC-derived HSCs in StemMACS HSC expansion medium, 1X STF for 21 days resulted in expansion of CD43+, CD34+, CD45+ HSCs. Table S1. Primer sequences used for RT-PCR. Table S2. Differentiation capacity of healthy donor and GS-2 BM-MSCs. For adipogenic differentiation, MSCs were cultured in DMEM-LG, supplemented with 10% FBS, 1 μM dexamethasone, 60 μM indometacin, 500 μM 3-isobutyl-1-methylxanthine and 5 μg/mL insulin. After 3 weeks, cells were stained with 2 mg/mL Oil Red O (ORO, Sigma-Aldrich O0625). ORO dye was extracted from the cells using %2 Igepal and measured at 496 nm on a microplate reader. For osteogenic differentiation, MSCs were cultured in DMEM-LG, 10% FBS, 100 nM dexamethasone, 10 mM beta-glycerophosphate and 0,2 mM L-ascorbic acid. Calcium levels were measured using the Quantichrom Calcium Analysis kit (BioAssay Systems, DICA-500). Table S3. Immunophenotype of healthy donor and GS-2 BM-MSCs. MSCs from healthy donors and GS-2 patients were generally positive for MSC-specific surface antigens (CD29, CD44, CD73, CD90, CD105 and CD166) and negative for hematopoietic and endothelial cell markers. No significant differences were found in expression of cell surface antigens by MSCs between the healthy donors and GS-2 patients. [file 13287_2021_2364_MOESM1_ESM.docx]

**Supplemental data**

**Figure S1. Karyotype analysis of healthy donor and GS-2 iPSC clones.**


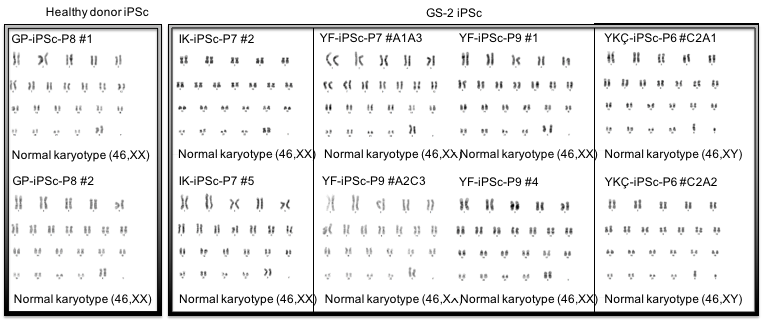


For karyotyping, iPSC cultures were trypsinized and treated with a hypotonic salt solution. 10 metaphases were captured and analysed. Shown here are the karyotypes of a healthy donor (left, GP) and the 3 GS-2 patient-derived iPSCs (IK, YF, YKÇ).

**Figure S2.**

**
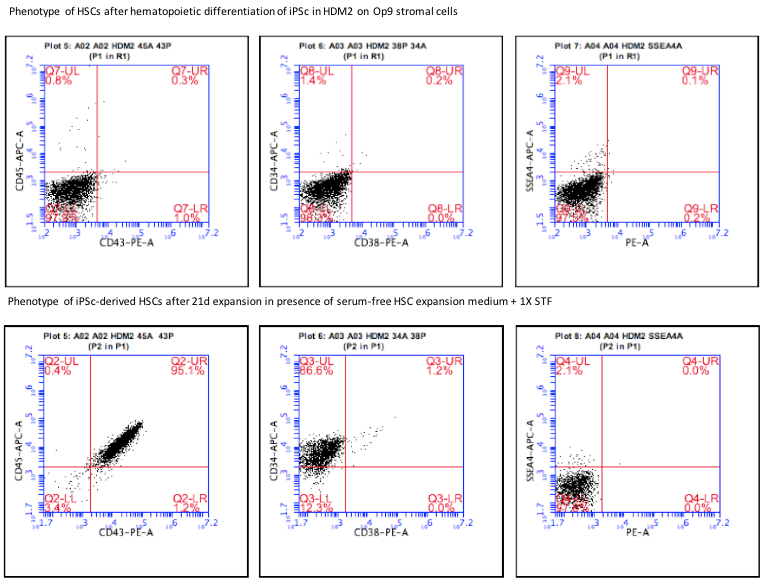
**

Upper panel: iPSCs were co-cultured with Op9 cells in presence of HDM2 medium (StemMACS HSC expansion medium, 1X STF, 10 µg/mL rhBMP-4), resulting in low level expression of CD34. Lower panel: further expansion of iPSC-derived HSCs in StemMACS HSC expansion medium, 1X STF for 21 days resulted in expansion of CD43+, CD34+, CD45+ HSCs.

**Table S1. Primer sequences used for RT-PCR**

| **Gene Name** | **Forward primer sequence** | **Reverse primer sequence** |
| --- | --- | --- |
| *B2M* | CCGTGTGAACCATGTGACTTT | CCTCCATGATGCTGCTTACA |
| *SOX2* | ATGGGTTCGGTGGTCAAGT | GGAGGAAGAGGTAACCACAGG |
| *NANOG* | ATGCCTCACACGGAGACTGT | CTGCAGAAGTGGGTTGTTTG |
| *OCT4* | GCAAAACCCGGAGGAGTC | TCCCAGGGTGATCCTCTTCT |

**Table S2. Differentiation capacity of healthy donor and GS-2 BM-MSCs**

| BM-MSCs | Adipogenic differentiation  (ORO, mg/mL) | Osteogenic differentiation  (Calcium, mg/dL) |
| --- | --- | --- |
| Healthy (n=7) | 0,47 ± 0,36 | 10,83 ± 5,68 |
| GS-2 (n=4) | 0,59 ± 0,97 | 19,29 ± 7,38 |

# For adipogenic differentiation, MSCs were cultured in DMEM-LG, supplemented with 10% FBS, 1 μM dexamethasone, 60 μM indometacin, 500 μM 3-isobutyl-1-methylxanthine and 5 μg/mL insulin. After 3 weeks, cells were stained with 2 mg/mL Oil Red O (ORO, Sigma-Aldrich O0625). ORO dye was extracted from the cells using %2 Igepal and measured at 496 nm on a microplate reader. For osteogenic differentiation, MSCs were cultured in DMEM-LG, 10% FBS, 100 nM dexamethasone, 10 mM beta-glycerophosphate and 0,2 mM L-ascorbic acid. Calcium levels were measured using the Quantichrom Calcium Analysis kit (BioAssay Systems, DICA-500).

**Table S3. Immunophenotype of healthy donor and GS-2 BM-MSCs**

| **BM-MSCs** | **CD29**  **(%)** | **CD44**  **(%)** | **CD73**  **(%)** | **CD90**  **(%)** | **CD105 (%)** | **CD34**  **(%)** | **CD31**  **(%)** | **CD166**  **(%)** | **HLA-DR (%)** |
| --- | --- | --- | --- | --- | --- | --- | --- | --- | --- |
| **Healthy**  **(n=5)** | 96,5 ± 4,2 | 98,0 ±  2,7 | 97,5 ±  4,8 | 99,4 ±  0,6 | 87,5 ±  25,6 | 0,6 ±  0,3 | 0,7 ±  0,6 | 93,6 ±  4,1 | 10,8 ±  6,6 |
| **GS-2**  **(n=4)** | 99,0 ± 0,7 | 98,7 ±  1,0 | 99,5 ±  0,6 | 98,9 ±  1,0 | 99,6 ±  0,5 | 0,5 ±  1,1 | 3,8 ±  6,4 | 73,4 ±  2,3 | 8,9 ±  10,0 |

MSCs from healthy donors and GS-2 patients were generally positive for MSC-specific surface antigens (CD29, CD44, CD73, CD90, CD105 and CD166) and negative for hematopoietic and endothelial cell markers. No significant differences were found in expression of cell surface antigens by MSCs between the healthy donors and GS-2 patients.
